# Ulnar Shortening Osteotomy and Reduction Assisted by 3D-Printed Guide Plate and Predrilling Technique

**DOI:** 10.1055/a-2646-9394

**Published:** 2025-07-14

**Authors:** Wu Xing, Zhao Jianyong, Zhang Zhisheng, Wang Wei

**Affiliations:** 1 Second Department of Hand and Microsurgery, Hebei Cangzhou Hospital of Integrated Traditional Chinese Medicine and Western Medicine, Cangzhou, Hebei, China; 2 Hebei Key Laboratory of Integrated Traditional and Western Medicine in Osteoarthrosis Research, Cangzhou, Hebei, China

**Keywords:** ulnar shortening osteotomy, 3D-printed guides, ulnar impaction syndrome, predrilling technique, cost-efficiency

## Abstract

**Background:**

Ulnar shortening osteotomy (USO) is a common surgical procedure for ulnar impaction syndrome. However, some hospitals lack specialized USO plates, or patients decline them due to high costs. The freehand technique for USO lacks precision; therefore, we utilized 3D-printed guide plates to assist in performing USO and fixation with standard ulnar locking plates.

**Materials and Methods:**

A retrospective case series of 87 patients was conducted using 3D-printed guide plates to achieve precise USO and fixation with standard ulnar locking plates. Primary outcomes included the patient-rated wrist evaluation (PRWE), disabilities of the arm, shoulder, and hand (DASH) questionnaire, and a custom patient satisfaction survey. Secondary outcomes included implant removal due to irritation and other complications.

**Results:**

The mean postoperative functional scores were 26 (standard deviation [SD]: 30) for PRWE and 21 (SD: 26) for DASH. Seventy-five patients reported satisfaction with the procedure. Thirty-six patients underwent implant removal due to irritation, with no plate fractures observed. One patient experienced nonunion, which healed after bone grafting, yielding a union rate of 98.9%.

**Conclusion:**

This technique offers precise shortening and improved osteotomy angulation accuracy while increasing the contact area at the osteotomy site—theoretically reducing the nonunion rate. It is particularly suitable for hospitals without access to specialized USO plates.

Ulnar impaction syndrome (UIS) is a common degenerative wrist disorder, with a prevalence of 10 to 20% among patients with ulnar positive variance. 1 2 Ulnar shortening osteotomy (USO) is a widely adopted surgical intervention for UIS, aiming to reduce ulnocarpal joint loading by shortening the ulna. Osteotomy techniques include transverse, oblique, and step-cut methods, each offering distinct biomechanical stability and fixation feasibility. 3 Internal fixation with plates or screws is typically employed to stabilize the bone during healing. Clinical studies have demonstrated high success rates for this procedure, with significant pain relief and functional improvement. 4 Advances in plate design have further enhanced surgical outcomes, reducing complication rates and accelerating recovery. 5

However, dedicated USO fixation plates remain cost-prohibitive and underutilized, particularly in resource-limited settings, posing a barrier to widespread adoption.

The use of conventional locking plates without specialized guides presents technical challenges. Freehand osteotomy may result in nonparallel resection surfaces, complicating reduction and stable fixation, especially for less-experienced surgeons. 3

To address these limitations, our protocol integrates 3D-printed guide plates with conventional ulnar locking plates and predrilling techniques, offering a cost-effective solution with enhanced precision. The 3D-printed guides facilitate parallel and accurate osteotomies aligned with preoperative plans, improving control over shortening length and angulation. 3 Additionally, predrilling streamlines reduction and fixation while enhancing structural stability to promote union. 4 This hybrid approach combines economic accessibility with technical reliability, demonstrating broad applicability across diverse clinical environments.

## Surgical Technique

(1) Preoperative planning and precision modeling: Preoperative evaluation is essential to confirm the diagnosis of UIS and associated injuries. The process begins with a detailed medical history, focusing on pain patterns and functional impairments. Physical examination should include ulnocarpal stress testing through maximal ulnar deviation and pronation with wrist flexion/extension to provoke pain. For a comprehensive assessment, standard wrist X-rays, including anteroposterior and lateral views, as well as pronated grip views, are utilized to evaluate ulnar variance and chondromalacia. Additionally, MRI helps detect lunate cartilage damage and lunotriquetral ligament tears.

**Fig. 1:**
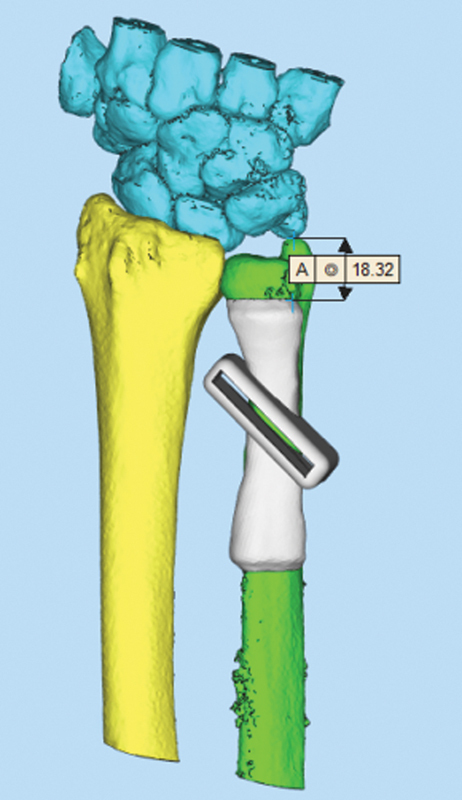
Using preoperative CT scan data, models of the distal ulna, radius, and wrist joint were created, along with the design of an osteotomy guide plate model.

**Fig. 2:**
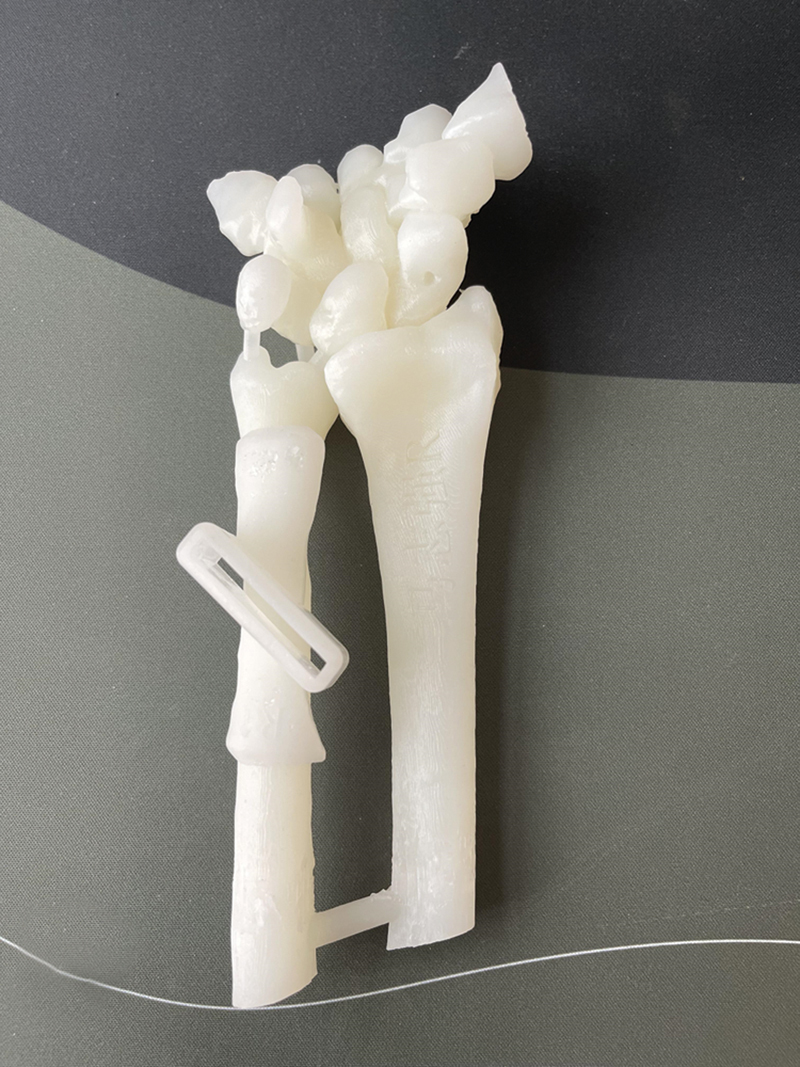
Based on the designed models, the 3D printer produced the physical models. Surgical procedure and technical key points.

The core component of our technique involves high-resolution CT scans integrated with 3D printing technology. Thin-slice CT data of the distal ulna, radius, and wrist joint are obtained and processed using 3D reconstruction software to design a personalized osteotomy guide. This guide incorporates a 45-degree angle relative to the long axis of the ulna and is positioned 5 to 7 cm proximal to the ulnar styloid. The resulting printed guide and bone models facilitate precise surgical simulation and planning ( Figs. 1 and 2 ). For manufacturing, UV-curable liquid photosensitive resin is used, which has been validated for short-term application in the human body. To ensure patient safety, the material undergoes standard sterilization processes before intraoperative use.

(2) Surgical procedure and technical key points: The surgical approach comprises two major phases: arthroscopic exploration followed by osteotomy and fixation.

In the arthroscopic exploration phase, the patient is positioned supine with the forearm secured in a wrist traction tower. The ulnocarpal joint is thoroughly explored through three to four portals. During this exploration, the central tear of the triangular fibrocartilage complex (TFCC), lunate cartilage flaps, and partial lunotriquetral ligament injuries are identified and debrided, which confirms the diagnosis of impaction.

For the osteotomy and fixation phase, an 8 cm longitudinal incision is made over the distal third of the ulna. Careful dissection is performed in the extensor/flexor carpi ulnaris interval to expose the ulna, followed by subperiosteal dissection to prepare for plate placement and osteotomy.

**Fig. 3:**
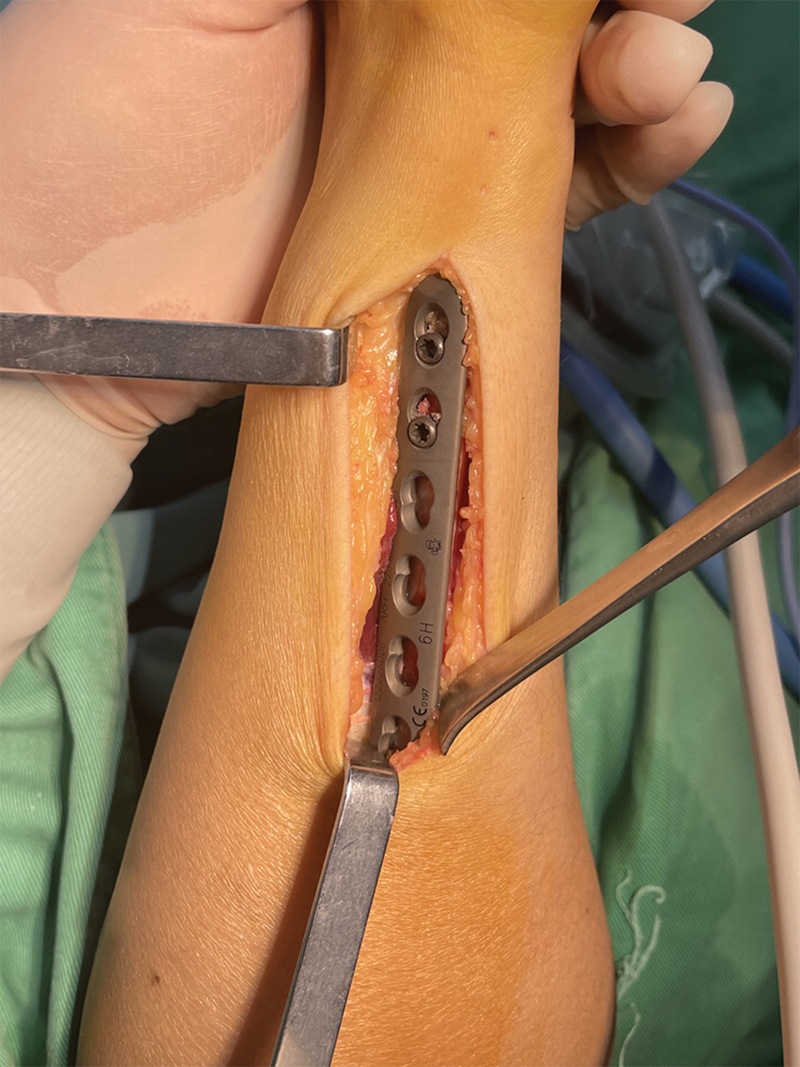
Using locking sleeve, drill two distal holes through the plate and then insert locking screws.

**Fig. 4:**
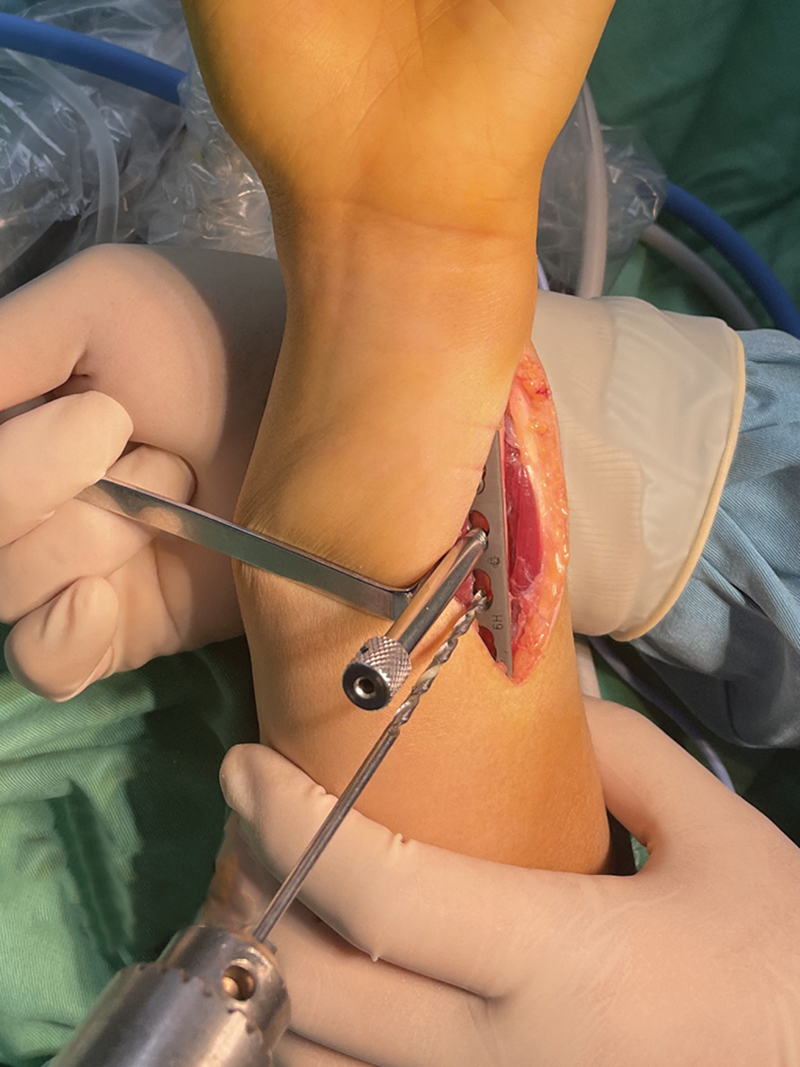
At the third hole location, attach the locking sleeve. Parallel the sleeve drill a hole at the fourth tension screw (nonlocking) position.

**Fig. 5:**
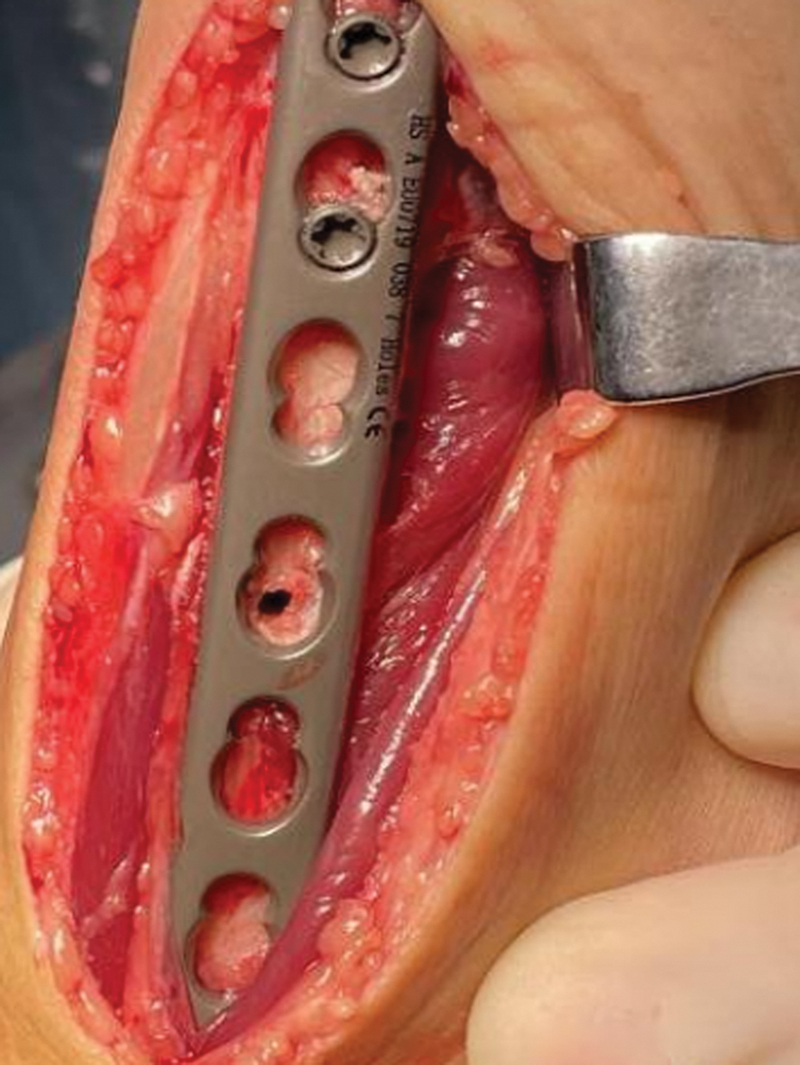
This hole will automatically align with the position of the locking screw hole after completing the osteotomy.

The plate prepositioning process begins with placement of a Zhejiang Kehui ZSQ24-S type 3.5 mm locking compression plate (narrow, LC-LCP) on the volar side of the ulna, positioned 1 to 2 cm distal to the ulnar head. Using a locking drill guide, two distal holes are drilled, and locking screws are inserted to secure the distal portion of the plate. The locking drill guide is then utilized to drill parallel holes for a fourth tension screw (nonlocking hole), after which the plate is temporarily removed ( Figs. 3 , 4 , 5 ).

**Fig. 6:**
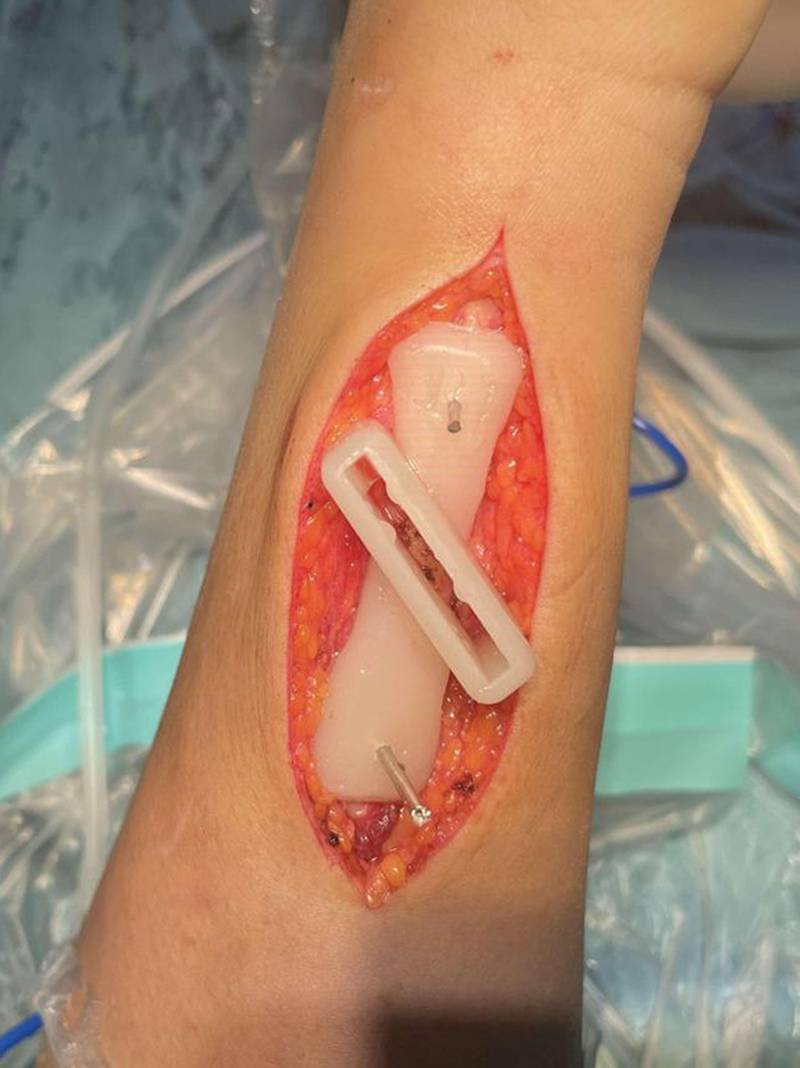
Installation of 3D-printed guide plate and osteotomy.

Following plate prepositioning, the sterilized 3D guide is fixed 2 cm proximal to the ulnar styloid to assist with the osteotomy. An oscillating saw is guided through the guide slot to perform a precise osteotomy. Throughout the cutting process, the saw blade is maintained firmly against both the distal and proximal surfaces of the guide to ensure maximum parallelism. While this approach significantly enhances parallelism compared with freehand techniques, it should be noted that absolute parallelism cannot be guaranteed due to the inherent flexibility of the saw blade ( Fig. 6 ).

**Fig. 7:**
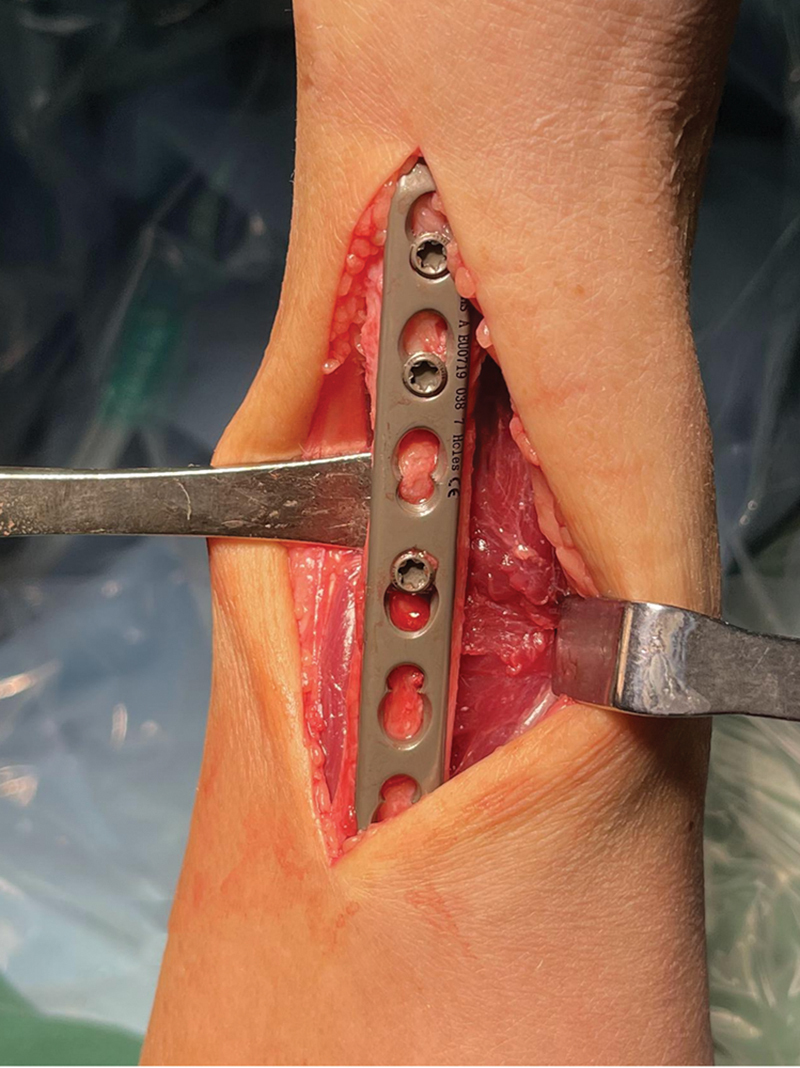
After achieving the proper reduction, the predrilled hole (located at the proximal part of the ulna) should align perfectly with the locking screw hole on the plate.

**Fig. 8:**
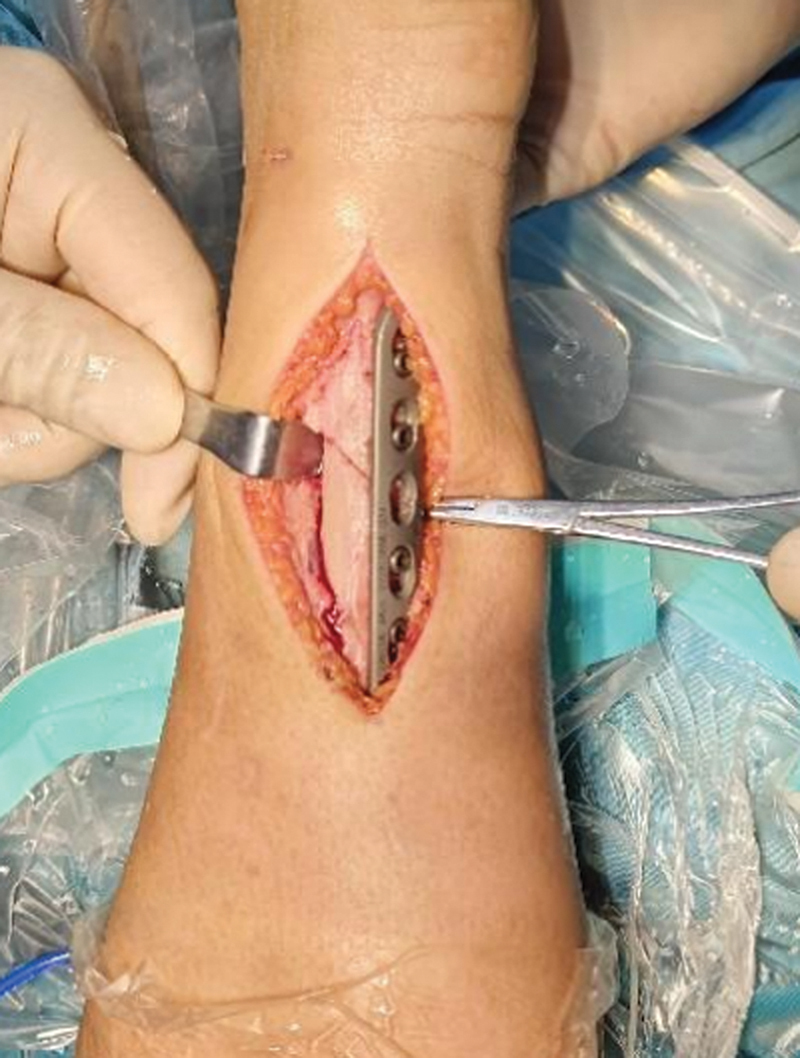
Postfixation immediate imaging.

For the reduction and fixation stage, the plate is first attached by inserting two distal locking screws. The osteotomy site is then anatomically reduced to restore normal ulnar alignment, with bone clamps applied to maintain this alignment during fixation. Upon achieving proper reduction, the fourth predrilled hole on the proximal ulna should align perfectly with the locking screw hole on the plate ( Fig. 7 ). While the reduction clamps secure the alignment, a locking screw is inserted into the fourth locking hole. Fluoroscopy confirmation ensures adequate ulnar shortening and correct osteotomy reduction. After verification of proper reduction and alignment, the remaining locking screws are inserted to secure the plate. Although we employ a seven-hole plate, typically two screws are placed distally and three proximally, with the two central holes left empty ( Fig. 8 ). The amount of shortening achieved with this technique typically ranges from 3 to 4 mm, which adequately addresses most patients with UIS. Our series did not include patients with excessive ulnar positive variance, so individualized adjustment of the shortening amount was not necessary.

(3) Postoperative management and rehabilitation: Postoperative care follows a progressive rehabilitation protocol designed to optimize healing and functional recovery. During the initial immobilization phase (0–4 weeks), patients are maintained in a short-arm cast for the first 2 weeks, after which sutures are removed. This is followed by a transition phase (4–6 weeks) where patients switch to a removable splint and begin gentle range-of-motion (ROM) exercises to prevent stiffness while protecting the healing osteotomy site. The functional restoration phase begins after 6 weeks, when radiographic evidence of bone healing typically appears (mean: 2.6 months). At this stage, patients progress to more advanced exercises with gradually increasing load and full wrist mobility training. Throughout the rehabilitation process, neurovascular status is carefully monitored, and outcomes are assessed using HAND20 scores and grip strength tests. Target recovery benchmarks include achieving greater than 90% dorsiflexion compared with the contralateral side and more than 50% reduction in pain levels.

## Materials and Methods

Between 2019 and 2024, we conducted a retrospective analysis of 87 patients (48 males and 37 females) who underwent USO for UIS using our 3D-printed guide plates combined with conventional ulnar locking plates and predrilling techniques.

Our study included patients experiencing persistent ulnar-sided wrist pain that worsened with ulnar deviation and had not responded to at least 6 months of conservative therapy. All participants had confirmed a UIS diagnosis supported by radiographic evidence. We excluded patients with concomitant TFCC foveal tears or distal radioulnar joint (DRUJ) instability, as well as those with ulnar variance exceeding 6 mm or presenting with Madelung deformity.

For preoperative assessment, all patients underwent bilateral wrist radiographs in neutral position to measure ulnar variance, defined as the length difference between the distal radial sigmoid notch and the most distal aspect of the ulnar dome. Positive ulnar variance was diagnosed when the ulnar dome extended distally beyond the ulnar corner of the sigmoid notch. We determined that cases with ulnar variance less than 5 mm were suitable candidates for this procedure, while sigmoid notch morphology did not influence our patient selection.

The study population had a mean age of 43.2 years, ranging from 16 to 75 years. All participants underwent preoperative wrist arthroscopy to evaluate TFCC integrity and articular surfaces before proceeding with the osteotomy. Throughout the follow-up period, we systematically documented any postoperative complications, including complex regional pain syndrome.

To evaluate outcomes, we employed multiple assessment methods, including functional outcomes measured via the QuickDASH questionnaire and patient-rated wrist evaluation (PRWE). Patient satisfaction was assessed using a four-point Likert scale where patients could indicate whether they were very satisfied, satisfied, somewhat dissatisfied, or dissatisfied with their surgical results.

## Results

The study included 87 patients with UIS. Preoperative radiographic evaluation revealed a mean positive ulnar variance of 3.7 mm (range: 2.1–5.8 mm). Postoperative measurements demonstrated significant correction to a mean −1.9 mm ulnar variance (range: −3.5 to 0.2 mm), achieving an average shortening of 3.5 mm (range: 2.8–4.7 mm).

Bony union was achieved in all but one patient (union rate: 98.9%), with a mean healing time of 11.4 weeks (standard deviation [SD]: ± 2.3 weeks). At the 6-month follow-up, functional outcomes were as follows:

- PRWE: Mean score 26 (SD: ± 30).
- Disabilities of the arm, shoulder, and hand (DASH): Mean score 21 (SD: ± 26).

Seventy-five patients (86.2%) reported satisfaction with surgical outcomes. Thirty-six patients (41.4%) required implant removal due to personal preferences, with no cases of plate or screw fracture observed. One patient developed nonunion, which resolved after bone grafting (final union rate: 98.9%). During the 24-month follow-up period, no cases of DRUJ instability or recurrent nonunion were documented.

## Discussion

### Union Rates and Surgical Precision

Historically, USO has been associated with nonunion rates as high as 13%, primarily due to challenges in osteotomy stability and precision. 6 In our study, the nonunion rate was only 1.1%, aligning with recent reports of high union rates achieved through advanced surgical techniques. 7 8 The single case of nonunion successfully healed following autologous iliac crest bone grafting. This low nonunion rate may be attributed to the use of 3D-printed guides, which ensured highly accurate osteotomies. The predrilling technique further improved reduction stability and reduced the mean fixation time to 22 minutes, consistent with prior studies demonstrating the precision benefits of predrilling. 9 Additionally, 3D-printed guides minimized irregular osteotomy surfaces, which are known to compromise stability and impair healing. 10

### Functional Outcomes

All patients in our cohort showed significant improvements in pain scores, ROM, grip strength, and modified Mayo wrist scores, regardless of postoperative ulnar variance (negative, neutral, or positive). These findings corroborate previous studies indicating that USO effectively alleviates pain and enhances function irrespective of specific ulnar variance magnitudes. 11 12 Notably, all patients returned to work within a mean of 4 months postoperatively, underscoring the technique's capacity to facilitate rapid recovery. Our functional outcomes parallel those of studies employing advanced fixation protocols. 13 14

### Cultural and Economic Considerations

A striking observation was the 41% implant removal rate, reflecting a cultural preference among Chinese patients to remove hardware postrecovery. This trend aligns with Xu et al's 15 findings in East Asian populations, where the perception that foreign materials disrupt natural healing processes drives high removal rates. Consequently, cultural factors must be prioritized in treatment planning, particularly in regions with elevated hardware removal demands. 11

Economically, 3D-printed guides demonstrated significant cost-effectiveness. The average cost of a standard locking plate was USD 120, while 3D-printed guides cost only USD 67. This represents a substantial reduction compared with traditional specialized osteotomy plates, which remain prohibitively expensive in low-resource settings. 16 The affordability of 3D printing enhances access to USO in resource-limited environments. 17 The choice of the 3.5 mm plate is appropriate for most Chinese male patients, although it may be slightly oversized for female patients. However, due to medical insurance policy limitations in China, alternative plate options are not available.

## Conclusion

Our findings demonstrate that USO using a seven-hole 3.5 mm standard locking plate combined with 3D-printed guides and predrilling is an effective, minimally invasive, and cost-efficient treatment for symptomatic UIS. The technique enables precise osteotomies, rapid union, and functional recovery without costly specialized instrumentation. However, long-term outcome studies are needed to validate durability, alongside efforts to optimize the protocol across diverse cultural and economic contexts.

Future research should focus on scaling 3D-printed guide systems in low-resource settings and developing cost-effective, customizable solutions for orthopedic surgery. Additionally, investigating cultural influences on outcomes—particularly implant removal—will provide critical insights for optimizing postoperative care.
